# Iodine status during pregnancy and at 6 weeks, 6, 12 and 18 months post‐partum

**DOI:** 10.1111/mcn.13050

**Published:** 2020-06-29

**Authors:** Inger Aakre, Marianne Sandsmark Morseth, Lisbeth Dahl, Sigrun Henjum, Marian Kjellevold, Vibeke Moe, Lars Smith, Maria Wik Markhus

**Affiliations:** ^1^ Department of Seafood, Nutrition and Environmental State Institute of Marine Research Bergen Norway; ^2^ Department of Nursing and Health Promotion, Faculty of Health Sciences OsloMet—Oslo Metropolitan University Oslo Norway; ^3^ Department of Psychology University of Oslo Oslo Norway

**Keywords:** breast feeding, iodine intake, post‐partum period, pregnancy, toddlers, urinary iodine concentration

## Abstract

Iodine deficiency during pregnancy and in the post‐partum period may lead to impaired child development. Our aim is to describe iodine status longitudinally in women from pregnancy until 18 months post‐partum. Furthermore, we explore whether iodine status is associated with dietary intake, iodine‐containing supplement use and breastfeeding status from pregnancy until 18 months post‐partum. We also assess the correlation between maternal iodine status 18 months post‐partum and child iodine status at 18 months of age. Iodine status was measured by urinary iodine concentration (UIC) during pregnancy (*n* = 1,004), 6 weeks post‐partum (*n* = 915), 6 months post‐partum (*n* = 849), 12 months post‐partum (*n* = 733) and 18 months post‐partum (*n* = 714). The toddlers' UIC was assessed at 18 months of age (*n* = 416). Demographic variables and dietary data (food frequency questionnaire) were collected during pregnancy, and dietary data and breastfeeding practices were collected at all time points post‐partum. We found that iodine status was insufficient in both pregnant and post‐partum women. The UIC was at its lowermost 6 weeks post‐partum and gradually improved with increasing time post‐partum. Intake of milk and use of iodine‐containing supplements significantly increased the odds of having a UIC above 100 μg/L. Neither the mothers' UIC, vegetarian practice, nor exclusion of milk and dairy products were associated with the toddlers UIC 18 months post‐partum. Women who exclude milk and dairy products from their diets and/or do not use iodine‐containing supplements may be at risk of iodine deficiency. The women possibly also have an increased risk of thyroid dysfunction and for conceiving children with nonoptimal developmental status.

**Key Messages**

- Iodine status was insufficient in pregnant women and at 6 weeks and 6‐, 12‐ and 18‐months post‐partum in a country‐representative sample from the LiN cohort.
- The iodine status was at its lowermost in women 6 weeks post‐partum, and gradually improved with increasing time post‐partum.
- Intake of milk and use of iodine‐containing supplements significantly increased the OR of having an UIC above 100 μg/L. Breastfeeding gave significantly reduced the OR of having UIC above 100 μg/L.
- The mothers' iodine status, vegetarian practice, exclusion of milk and dairy products were not associated with the toddler's iodine status at 18 months post‐partum.

## 1 INTRODUCTION

Iodine is a micronutrient and an essential component of the thyroid hormones. Thyroid hormones regulate a wide range of cellular and physiological functions and are essential for normal child growth and development. Therefore, thyroid hormone insufficiencies during fetal and neonatal growth may lead to impaired physical and mental development (Zimmermann, 2011). Iodine is present in relatively few foods, and iodized salt is the most important source of iodine globally (WHO, 2007). In the Norwegian population, lean fish, dairy products and eggs are the major dietary iodine sources due to naturally occurring levels in the sea and iodine fortification of animal fodder, respectively. Furthermore, iodine fortification of salt is not mandatory, and the permitted level of supplementation is low (5 μg/g; Carlsen, Andersen, Dahl, Norberg, & Hjartåker, 2018; Dahl, Johansson, Julshamn, & Meltzer, 2004). During the last decade, intake of fish and milk has decreased in Norway (The Norwegian Directorate of Health, 2018). Iodine deficiency is prevalent in Europe, especially among pregnant women (Iodine Global Network, 2018). Recent studies from Norway confirm insufficient iodine intake, especially in young, pregnant and lactating women (Dahl et al., 2018; Henjum, Aakre, et al., 2018; Henjum, Brantsæter, et al., 2018; Henjum et al., 2017). Additionally, two studies have found an association between suboptimal maternal iodine intake during pregnancy and impaired child development, especially in the area of language skills (Abel et al., 2017; Markhus et al., 2018). Infants are vulnerable to iodine deficiency due to their small thyroidal iodine stores, high thyroxine turnover and high iodine requirements per body weight, compared with other age groups (Brown, 2009; Delange, 1998). Breastfed infants depend on sufficient maternal iodine intake for optimal growth and development (Henjum et al., 2017).

In pregnancy, iodine requirements are elevated due to increased thyroid hormone synthesis, transfer of iodine to the fetus, and increased glomerular filtration, resulting in increased urinary losses (Dafnis & Sabatini, 1992; Glinoer, 2007). Iodine requirements are also increased during lactation, as iodine is secreted into breast milk and serves as a crucial iodine source to the breastfeeding infant (Andersson, De Benoist, Delange, & Zupan, 2007). The Nordic Nutrition Recommendations (NNR) recommend an iodine intake of 175 μg/day, whereas the World Health Organization (WHO) recommend 250 μg/day for pregnant women (NNR, 2014; WHO, 2007).

WHO recommend use of spot urine as an easy and cost‐efficient method for assessing iodine status in population groups (WHO, 2007). A median urinary iodine concentration (UIC) between 150 and 249 μg/L is considered adequate in pregnant women. The lowest limit in the interval, 150 μg/L, is based on the recommended nutrient intake (RNI) of 250 μg/day, assuming 90% of the ingested iodine is excreted in urine and a median urine volume of 1.5 L (Andersson et al., 2007). Using the same assumptions for the Nordic recommendations (175 μg/day), this would correspond to a median UIC of 105 μg/L. There are challenges associated with the use of UIC as a marker in lactating women, because about 40–50% of the ingested iodine is secreted through breast milk (Laurberg & Andersen, 2014), and thus, BMIC should also ideally be measured (Dold et al., 2017). A median UIC > 100 μg/L is considered adequate in lactating women. Iodine concentration in urine may vary considerably among individuals, both from day to day and between days, because iodine is found in relatively few foods (Andersen, Karmisholt, Pedersen, & Laurberg, 2008; Rasmussen, Ovesen, & Christiansen, 1999). UIC also varies with hydration level (Vejbjerg et al., 2009; Zimmermann, 2008). Due to this anticipated large intraindividual variability, according to WHO, UIC should be assessed at a group level (WHO, 2007).

We present country‐representative data from the Little in Norway (LiN) cohort study. The objective is to describe UIC during pregnancy and at 6 weeks and 6, 12 and 18 months post‐partum, and to assess and track the stability of UIC measurements over time. Furthermore, we will explore whether UIC is associated with dietary intake, supplement use and breastfeeding status from pregnancy until 18 months post‐partum. We also assess the correlation between maternal UIC at 18 months post‐partum and child UIC at 18 months of age.

## 2 METHODS

### 2.1 Study design and participants

This paper is based on data from the LiN study, a prospective cohort conducted from September 2011 until November 2014 (Moe et al., 2019). Pregnant women were enrolled from nine primary health clinics across all four Norwegian health regions, representing both urban and rural regions. The gestational age at enrolment varied from 9 to 34 weeks, and the women were followed until 18 months post‐partum. The pregnant women were approached by midwives at primary health clinics with a study invite. Further details regarding study recruitment and attrition has been reported by Fredriksen et al. (Fredriksen, von Soest, Smith, & Moe, 2017, 2018; Moe et al., 2019). This study uses information from five data collection waves: pregnancy, 6 weeks post‐partum, 6 months post‐partum, 12 months post‐partum and 18 months post‐partum. All women who submitted a urine sample at one or several data collection waves were included. Details regarding inclusion of participants in each wave can be found in Appendix S1. UIC in pregnancy have been published previously (Dahl et al., 2018; Markhus et al., 2018). Data on demographic variables, smoking habits, self‐reported thyroid medication and maternal anthropometrics were only collected during the first wave (pregnancy), dietary habits were collected at all waves, and data regarding breastfeeding status were collected at each wave post‐partum (at 6 weeks and 6, 12 and 18 months).

Iodine status was also measured among the toddlers (*n* = 416) of participating women at 18 months of age. Data on iodine status in toddlers at 18 months of age were included in order to assess correlation between mothers' and toddlers' iodine status. The toddlers' iodine status has previously been published (Aakre et al., 2018).

### 2.2 Urinary iodine concentration

Spot urine samples were collected during pregnancy and at 6 weeks and 6, 12 and 18 months post‐partum. A nonfasting urine sample was obtained from the women upon consultations. If forgotten, the urine sample was collected at site. Samples were collected in plastic tubes, transferred to test tubes marked with gestational week, date and time of the day. Urine from the toddlers were collected using Uricol collection pack (Sterisets International B.V., SteriSets GP Supplies, Newcastle Urine Collection Pack, UK). The urine was extracted from the pad with a syringe and transferred to CryoTubes (CryoTubes™ Vials, Nunc A/S, Roskilde, Denmark). The samples were stored at −20°C, pending analysis at the Institute of Marine Research (IMR) in Bergen. The UIC was determined by inductively coupled plasma mass spectrometry (ICP‐MS). Description of the analytical procedures has previously been published (Dahl et al., 2018). The accuracy of the results was verified with certified reference material; Seronorm Trace Elements Urine (Nycomed Pharma, Asker, Norway) with certified iodine content of 84 (range 72–96 μg/L) and 304 μg/L (range 260–348 μg/L).

### 2.3 Breastfeeding

Breastfeeding status and frequency were measured during all post‐natal data collection waves by the following two coded questions: ‘Does your child receive breast milk now’ and ‘How often does your child receive breast milk’.

### 2.4 Dietary intake

Information about dietary intake was collected at all data collection waves using a web‐based semiquantitative food frequency questionnaire (FFQ), originally developed to assess intake of seafood and omega‐3 fatty acids (Dahl, Mæland, & Bjørkkjær, 2011). The FFQ covered the last 3 months intake of iodine rich foods, such as seafood, milk, dairy products and eggs, in addition to some questions assessing intake of other main food groups, such as fruit and vegetables, bread and cereals, meat and meat products and vegetarian practice. This paper includes questions regarding intake of seafood as dinner, intake of milk and dairy products and intake of eggs, as these are considered the main dietary sources of iodine in Norway (Dahl & Meltzer, 2009). The FFQ has been described in detail elsewhere (Dahl et al., 2018). Data on frequency was dichotomized for regression analysis using the following cut‐offs: fish for dinner ≤1 time/week and ≥2 times per week; milk and dairy products ≤1 time/day and ≥2 times per day; eggs ≤3 times/week and ≥4 times per week. The FFQ included questions on supplement use. However, the questions were designed to comprise all types of dietary supplements and not iodine specifically. Therefore, all variables regarding supplements were manually checked by examining the package insert for the given brand in order to examine whether the supplement contained iodine or not and at what amount. Use of iodine‐containing supplements was transformed into three new variables: (1) use iodine‐containing supplements (yes/no), (2) frequency of use of iodine‐containing supplements and (3) amount of iodine in the supplements used. For most of the supplements, the specific brand was given in precoded options in the questionnaire. For multivitamin and mineral supplements, one option was to choose the category ‘other’. If this was done, and the specific brand name was not reported, it was scored as ‘yes’ on iodine‐containing supplement use, as most of the multivitamin and mineral supplements sold in Norway contain iodine. Vegetarian practice was coded ‘yes’ or ‘no’ and was not further predefined in the FFQ.

Estimated total iodine intake was based on UIC and calculated using the following formula: UIC (μg/L) × 0.0235 × weight in kg (prepregnancy weight was used for post‐partum women) (IOM, 2001).

### 2.5 Statistics

Data analyses were performed using the software IBM SPSS statistics version 25 (IBM SPSS Statistics Inc., Chicago, USA) and STATA version 15 (StataCorp, College Station, TX). Continuous data were presented as mean and standard deviation (SD) if normally distributed, and as median, 25 and 75 percentiles (p25–p75) if not normally distributed. Normality was assessed by inspection of normality plots.

Tracking analysis is used to assess the stability of a health behaviour or health outcome over time, or the ability of an individual to maintain their rank, including group membership over time (Mikkilä, Räsänen, Raitakari, Pietinen, & Viikari, 2005; J. W. Twisk, Kemper, & Mellenbergh, 1994; W. R. Twisk, Kemper, Mellenbergh, & van Mechelen, 1996). The cut‐offs for tracking coefficients are not universally defined because their magnitude may depend on the length of follow‐up and measurement error or variation in the variable being tracked (J. W. Twisk, 2013). The Cohen's weighted kappa coefficient is calculated based on movement between groups from one wave of measurement to the next. Whereas simple kappa analysis only considers a change in group membership, the weighted kappa assigns less weight to agreement if categories are further apart (Viera & Garrett, 2005). UIC for each data collection wave was divided into tertiles, and tertile membership was tracked from one wave to the next and from the first data collection wave to the last. Tracking was done using Cohen's weighted kappa (*κ*_w_), which accounts for the squared concordance of position among groups (Cohen, 1968). The weighted kappa was calculated using crosstab analysis and a script available from the IBM website (IBM, 2010). Stability is presented as percentage of participants remaining in their tertile membership and as percentage of participants that had a decrease and an increase in tertile since the previous time slot. The criteria from Landis and Koch (1977) were used to assess agreement, where a *κ*_w_ of 0.01–0.20 represents slight agreement, 0.21–0.40 fair agreement, 0.41–0.60 moderate agreement, 0.61–0.80 substantial agreement and 0.81–1.00 almost perfect agreement.

Associations between intake of iodine supplements or food groups (fish, milk and eggs) and UIC were assessed by generalized estimating equation (GEE) models with exchangeable covariance structure. A binary logistic model was chosen, and UIC was divided into <100 or ≥100 μg/L. This cut‐off was chosen as it corresponds well with the recommended iodine intake in the Nordic countries of 175 μg/day and has also been suggested by others (Abel et al., 2018; Markhus et al., 2018). The linearity of the associations between the logit of covariates and the dependent variable was checked by Lowess smooth function in Stata. Participants who reported the use of thyroid medication during pregnancy were excluded. Variables to be included in the models were chosen on a theory‐based approach and consisted of covariates known from previous studies to influence iodine status in pregnancy (Dineva et al., 2020; Henjum, Aakre, et al., 2018; Henjum et al., 2017). For iodine supplement use, an unadjusted model, then a model adjusted for maternal age, parity, breastfeeding status, time and time*supplement use was performed. For food groups, unadjusted models based on bivariate analysis, then models adjusted for the same covariates, iodine supplement use, time and a time*food group intake interaction were performed. During pregnancy, breastfeeding was set to ‘no’ for all participants.

Maternal cherecteristics and dietary habits at 18 months post‐partum and toddlers UIC at 18 months of age were tested for association. Mother's UIC, mother's BMI prepregnancy, mother's age, if mother excluded dairy products from her diet (yes/no), and mother's use of iodine‐containing supplements (yes/no) were used as independent variables in simple linear regression models. Standard residuals plots were examined and residuals above 3 or below −3 were removed from the models.

### 2.6 Ethical considerations

The trial complies with the Declaration of Helsinki and was commenced after approval by the Regional Committees for Medical and Health Research Ethics (2011/560 REK South‐East). Written informed consent was obtained from the participants who could withdraw from the study at any time.

## 3 RESULTS

Background characteristics collected during pregnancy are described in Table 1. The mean ± SD age of the women was 30.2 ± 4.8 years, and all geographical regions of Norway were represented.

**TABLE 1:** Characteristics of Norwegian women from the LiN study collected during wave one (*n* = 1,004)

Characteristics of pregnant women^a^	(*n* = 1,004)
Age (years)	30.2 ± 4.8
BMI (kg/m^2^)^b^	22.8 (20.8–25.3)
<18.5	31 [3.1]
18.5–24.9	554 [55.2]
≥25	216 [21.5]
Education level	
Primary and secondary school	32 [3.2]
High school	200 [19.9]
<4 years of university	360 [35.9]
≥4 years of university	412 [41.0]
Work situation	
Work full time	770 [76.7]
Work part time^c^	136 [13.5]
Student^d^	60 [6.0]
Unemployed	30 [3.0]
Geographic region	
Mid‐Norway	275 [27.4]
North Norway	144 [14.3]
Western Norway	218 [21.7]
Eastern Norway	367 [36.6]
Use medication for thyroid disorder	33 [3.3]
Use of iodine‐containing supplements	344 [34.3]
Number of live births	0.6 ± 0.8
Use tobacco daily	75 [7.5]

Values are presented as mean ± SD, median (p25–p75) and *n* [%]. Missing data: 203 missing from women's BMI and eight missing from work situation.

a Background characteristics were collected during wave 1.

b BMI before pregnancy.

c Six percent (*n* = 60) reporting a combination of work and studies.

d Part‐time or full‐time student.

**FIGURE 1:**
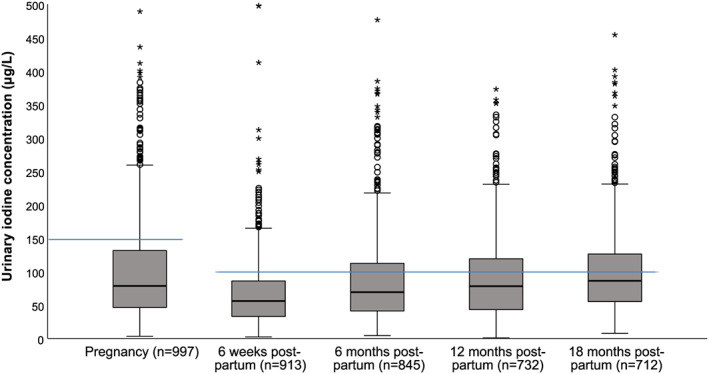
Urinary iodine concentration (UIC) at the different data collection waves. Cases with UIC > 500 μg/L were excluded from the figure. The blue lines indicate the cut‐off used by the WHO to assess adequate iodine status from UIC in pregnant (150 μg/L) and lactating women (100 μg/L). Boxes indicate the upper (p75) and lower (p25) quartile, with the thick black line giving the median (p50). The T‐bars indicate 1.5 × length of the box (interquartile range). The filled circles are outliers defined as a value > 1.5 length of the box. The asterisks are extreme outliers defined as a value > 3.0 length of the box

The median (p25–p75) UIC in pregnancy and at the different post‐partum stages are presented in Table 2. As seen in Table 2 and in Figure 1, the median UIC was lower at 6 weeks post‐partum (57 μg/L) than during pregnancy (79 μg/L). From 6 weeks post‐partum until 18 months post‐partum, the median UIC slowly increased to 87 μg/L at 18 months post‐partum. The same was seen for iodine intake estimated from the UIC, where the mean estimated intake was 140 μg/day in pregnancy, 87 μg/day 6 weeks post‐partum and 133 μg/day 18 months post‐partum. UIC was significantly different between lactating and nonlactating women only at 6 weeks post‐partum (*P* = 0.008). The median UIC, both during pregnancy and post‐partum was below the epidemiological criteria of adequate iodine nutrition from the WHO. A high share of the women had a UIC < 50 μg/L: 27% in pregnancy, 38% 6 weeks post‐partum, 27% 6 months post‐partum, 21% 12 months post‐partum and 15% 18 months post‐partum.

**TABLE 2:** Urinary iodine concentration (UIC) in μg/L among women in pregnancy and different post‐partum stages in the LiN study

	Pregnant women		6 weeks post‐partum		6 months post‐partum		12 months post‐partum		18 months post‐partum	
	*N*		*n*		*n*		*n*		*n*	
UIC (μg/L)	1,004	79 (47–132)	915	57 (34–86)	849	70 (42–113)	733	79 (44–120)	714	87 (56–127)
Estimated iodine intake^a^ (μg/day)	801	140 (77–228)	749	87 (51–196)	706	109 (63–176)	608	119 (68–189)	593	133 (84–196)
UIC lactating women (μg/L)	NA	NA	652	56 (33–83)	655	69 (41–113)	288	80 (47–118)	63	103 (73–146)
UIC nonlactating women (μg/L)	NA	NA	33	74 (44–108)	131	72 (46–114)	342	76 (41–123)	505	85 (54–129)
Estimated iodine intake nonlactating women (μg/day)	NA	NA	25	127 (79–227)	106	110 (61–184)	297	116 (66–190)	438	132 (84–193)

*Note*: Values for UIC and estimated iodine intake are given as median (p25–p75). NA: Not applicable. Differences in UIC between lactating and nonlactating women were done by Mann–Whitney *U* test, *P* = 0.008, 6 weeks pp; *P* = 0.634, 6 months pp; *P* = 0.352, 12 months pp; *P* = 0.058, 18 months pp.

a Iodine intake was estimate using the following formula UIC × 0.0235 × weight in kg (prepregnancy weight was used for post‐partum women) (IOM, 2001).

Tracking of tertile membership of UIC was fair and moderately stable from pregnancy to 6 weeks post‐partum (Cohen's *κ*_w_ 0.240) and from 6 weeks post‐partum to 6 months post‐partum (Cohen's *κ*_w_ 0.308; Table 3). Further, from 6 to 12 months post‐partum and from 12 to 18 months post‐partum, the tracking of tertile membership decreased and was very low (Cohen's *κ*_w_ 0.0276 and 0.0474). Finally, from pregnancy to 18 months post‐partum, the tertile membership had a slight agreement (Cohen's *κ*_w_ 0.157).

**TABLE 3:** Tracking of UIC from pregnancy until 18 months post‐partum among women in the LiN study

UIC tertile	Preg–6 weeks pp (*n* 915)			6 weeks to 6 months pp (*n* 849)			6–12 months pp (*n* 733)			12–18 months pp (*n* 714)			Preg–18 months pp (*n* 714)		
	Decrease	Stable	Increase	Decrease	Stable	Increase	Decrease	Stable	Increase	Decrease	Stable	Increase	Decrease	Stable	Increase
Low	NA	46.0	54.0	NA	51.3	48.7	NA	33.5	66.5	NA	36.0	64.0	NA	41.2	58.8
Middle	30.1	38.1	31.8	30.4	37.7	31.9	33.9	35.2	30.9	34.7	33.8	31.5	32.0	37.3	30.7
High	53.3	46.7	NA	51.5	48.5	NA	62.9	37.1	NA	63.8	36.2	NA	56.4	43.6	NA
*κ*_w_ ^a^	0.240			0.308			0.0276			0.0474			0.157		

Abbreviation: UIC, urinary iodine concentration.

a Tracking coefficient of Cohen's weighted kappa. A *κ*_w_ of 0.01–0.20 represents slight agreement, 0.21–0.40 fair agreement, 0.41–0.60 moderate agreement, 0.61–0.80 substantial agreement and 0.81–1.00 almost perfect agreement (Landis & Koch, 1977).

Associations between supplement use, and food group intake, and UIC are presented in Table 4. In adjusted models, the odds of being in the ≥100 μg/day UIC group were significantly higher (OR 1.82; CI: 1.35, 2.48; *P* < 0.001) for participants using iodine supplements compared with the group that had not used supplements and for participants consuming milk ≥2 times compared with ≤1 time per day (OR 1.64; CI: 1.12, 1.40; *P* = 0.010). In unadjusted analysis, there were significantly reduced odds of being in the ≥100 μg/day UIC group for participants who were breastfeeding (OR 0.62; CI: 0.54, 0.71; *P* < 0.001).

**TABLE 4:** Associations between supplement use or food group intake and urinary iodine concentration (UIC) among Norwegian women during pregnancy and up to 18 months post‐partum in the LiN study (*n* = 928)

	Model 1^a^			Model 2^b^		
	OR	95% CI	*P*	OR	95% CI	*P*
Diet						
Iodine supplements^c^	1.52	1.28, 1.81	<0.001	1.82	1.35, 2.48	<0.001
Fish^d^	1.12	0.95, 1.31	0.167	1.04	0.76, 1.42	0.808
Milk^e^	1.31	1.09, 1.58	0.004	1.64	1.12, 1.40	0.010
Eggs^f^	0.98	0.80, 1.22	0.885	1.11	0.75, 1.66	0.592
Baseline characteristics						
Maternal age	1.01	0.99, 1.02	0.402			
Parity	0.98	0.89, 1.09	0.729			
Breastfeeding^g^	0.62	0.54, 0.71	<0.001			

*Note*: Associations assessed by GEE models with exchangeable covariance matrix, outcome variable dichotomized (<100 vs. ≥100 μg/day), <100 μg reference category.

a Unadjusted estimates.

b Adjusted for baseline characteristics, time and time*supplement use/food group. Food groups also adjusted for supplement use

c Dichotomized (yes/no), ‘no’ as reference category.

d Categorized (fish for dinner ≤1 and ≥2 times per week), ≤1 time per week as reference category.

e Categorized (milk and dairy products ≤1 and ≥2 times per day), ≤1 time per day as reference category.

f Categorized (eggs ≤3 and ≥4 times per week), ≤3 times per week as reference category

g Dichotomized (yes/no), ‘no’ as reference category, set to ‘no’ for all participants during pregnancy.

The toddlers of the women in the study had their UIC measured at 18 months of age; these values have previously been published (Aakre et al., 2018). The median (p25–p75) UIC for the toddlers at 18 months of age was 129 (81–190) μg/L. A positive and non‐significant correlation was observed between mother and child UIC, Spearman's *rho* = 0.061 (*P* = 0.229), Appendix S2. The tertile agreement between mother and child UIC was very low, with a *κ*_w_ of 0.0312. The mothers' BMI, vegetarian practice and whether or not the mothers included dairy products in their diets were not significantly associated with the toddlers UIC in regression analyses (results not shown).

## 4 DISCUSSION

The main finding in this study was that median UIC was insufficient in women throughout pregnancy and up to 18 months post‐partum. The stability coefficient for UIC varied greatly and was at its lowermost between 6–12 and 12–18 months post‐partum. Only intake of milk and use of iodine‐containing supplements significantly increased the odds of having a UIC above 100 μg/L. Neither the mothers' UIC, vegetarian practice nor exclusion of milk and dairy products were associated with the toddlers UIC 18 months post‐partum.

The iodine intake was insufficient in pregnancy and during all post‐partum stages (6 weeks and 6, 12 and 18 months post‐partum), using the WHO epidemiological criteria for assessing iodine nutrition based on median UIC (WHO, 2007). Further, the median estimated iodine intake from the UIC was below the estimated average requirement (EAR) for pregnant and lactating women of 160 and 209 μg/day, respectively (IOM, 2001). Recent studies of pregnant and lactating women in Norway found similar levels of UIC as reported in this study, with a median UIC of 92 and 84 μg/L in pregnant women (Brantsæter et al., 2018; Henjum, Aakre, et al., 2018) and 68 μg/L in lactating women 2–28 weeks post‐partum (Henjum et al., 2017). The iodine status in this study was lowest at 6 weeks post‐partum, with a median UIC of 57 μg/L and 38% having a UIC < 50 μg/L. This is somewhat expected due to increased iodine loss through lactation (Leung, Pearce, & Braverman, 2011). Furthermore, the iodine status decrease with advancing gestation, as the renal loss and fetal demands of iodine increase (Dafnis & Sabatini, 1992; Glinoer, 2007). At the same time, the dietary compensation for these increased demands has been inadequate, possibly leading to a depletion of total body iodine stores. Another factor may be increased demands during the lactation period (Delange, 2007). The median UIC seemed to increase gradually until 18 months post‐partum, reaching a slightly higher level than during pregnancy. This was probably due to a decrease in breastfeeding during the post‐natal period. The same pattern was found in a study of Sudanese women living in an area of iodine deficiency, where UIC was measured in the first trimester of pregnancy and later at 3, 6 and 9 months post‐partum (Eltom, Eltom, Elnagar, Elbagir, & Gebre‐Medhin, 2000). An increase in UIC with time post‐partum was also seen in a randomized controlled trial with iodine‐containing supplements among Australian women, where UIC was measured from 1 week until 6 months post‐partum (Mulrine, Skeaff, Ferguson, Gray, & Valeix, 2010).

Tracking of tertile membership of UIC was fair and moderately stable from pregnancy to 6 months post‐partum. From 6 to 12 months post‐partum and from 12 to 18 months post‐partum, the tracking of tertile membership decreased and was very low. When the reproducibility of the outcome variable is low, tracking is expected also to be low (J. W. Twisk, 2003b). UIC is mainly determined by iodine intake from diet, and low tracking was anticipated due to the scarcity of foods rich in iodine and the speed with which iodine is excreted into urine (Rasmussen et al., 1999). If we had used 24‐h urine samples or spot samples taken at a standardized time point, this could possibly have resulted in a higher stability of iodine status. We do not know of any previous studies tracking UIC in the general population or among pregnant and breastfeeding women, but a comparison of stability coefficients between our study and women in other life stages would be of interest given the influence of pregnancy and breastfeeding on iodine status. For instance, the extremely low tracking from 6 to 12 months and 12 to 18 months could in part be due to a change in breastfeeding status for many of the women at various time points during this period of follow‐up. This might also explain why the stability coefficient was higher from pregnancy to 18 months than for the time period in between, even though tracking is generally expected to decrease with increasing time interval between measurements (J. W. Twisk, 2003a). Finally, tracking analysis based on tertile membership may be considered arbitrary compared with tracking based on objective cut‐offs related to health outcomes (J. W. Twisk, 2003b). In this study, data on thyroid function was not collected. However, future research assessing whether a stable high (tracking high vs. low) UIC has a positive influence on thyroid function would be of interest.

Intake of iodine‐containing supplements and intake of milk and dairy products ≥2 compared with ≤1 time per day significantly increased the odds of having UIC above 100 μg/L. Supplements and dairy products have been found to be the major iodine sources in other studies of pregnant and lactating women in Norway (Brantsæter, Abel, Haugen, & Meltzer, 2013; Henjum, Aakre, et al., 2018; Henjum et al., 2017). Similarly, in a study where pregnant women from three large cohorts in Europe were included, intake of milk and dairy products was the main dietary determinant of iodine status. Other dietary determinants were fish and shellfish and cereals (Dineva et al., 2020). Breastfeeding gave significantly reduced odds of having UIC above 100 μg/L in our study, which is not unexpected because large amounts of iodine are secreted into breast milk (Laurberg & Andersen, 2014). The lack of associations found between intake of fish or eggs and UIC may be due to how these categories of intake were defined. However, cut‐offs were deemed plausible based on intake frequencies in a typical Norwegian diet. A limitation in this regard, however, is that we only included fish for dinner, and not fish as spread, even though this is commonly consumed in Norway. Nevertheless, fish as dinner is consumed less frequently than milk and other dairy products, and thus, we do not necessarily expect to find an association with iodine measured in spot samples as such samples will contain iodine excreted only from the most recent intake. Therefore, we cannot exclude fish intake as a predictor of UIC based on our results. Using a binary outcome variable may also lead to loss of power in the model, especially when the outcome variable is highly skewed and there are few categories assigned (Taylor, West, & Aiken, 2006), which was the case in our study. However, these challenges are lessened by the large sample size. It should be noted that a binary outcome was chosen mainly due to the large intraindividual variation in UIC. The cut‐off used for the outcome variable (100 μg/L) is indicative of an insufficient iodine intake for pregnant women suggested by Abel et al. (2018) and Markhus et al. (2018). The same cut‐off was applied for breastfeeding women given the longitudinal nature of the data and has also been suggested by the WHO for assessing insufficient (<100 μg/L) and adequate (>100 μg/L) iodine status (WHO, 2007).

The mothers' UIC at 18 months post‐partum was not associated with the toddlers UIC at 18 months of age, nor were the mothers' dietary habits, such as exclusion of milk and dairy products or supplement use. Maternal iodine status is known to influence infant iodine status in breastfed children (Azizi & Smyth, 2009; Delange, 2007; Osei et al., 2016). However, at 18 months of age, breast milk is no longer a significant dietary component (only 7.5% were partially breastfed). Therefore, the mothers' iodine status is no longer likely to influence the children's status to a large degree. Exclusion of dairy products among mothers appeared not to influence the children's UIC, suggesting that this dietary practice was not transferred to the children.

### 4.1 Strengths and limitations

The longitudinal nature of the data provided a rare opportunity to assess iodine status several times during a vulnerable time period for iodine deficiency in women. Further, the study has a relatively large number of participants recruited from different geographical areas in Norway, which enable classification of iodine status based on median UIC in the population group (Vejbjerg et al., 2009). This study may not be representative in terms of socio‐economic status; however, previous studies have not evidenced an association between education, a major determinant of socio‐economic status, and UIC (Henjum, Aakre, et al., 2018; Henjum et al., 2017). The study also had limitations: Iodine status measured before pregnancy could have been beneficial in order to investigate how iodine status might have changed during the whole period up to 18 months post‐partum. Urine samples from the infants were only collected at 18 months, and unfortunately, no health outcomes were included in this study; it would have been of interest to examine if consistent low UIC might be associated with impaired thyroid function. Further, use of a dichotomized variable for intake of breast milk was a limitation. However, data on the amount of breast milk consumed were not collected because there was no feasible and valid method available in this study.

## 5 CONCLUSION

We found that pregnant and lactating women in a large study of Norwegian women had insufficient iodine status. The UIC was at its lowermost 6 weeks post‐partum and then gradually improved with increasing time post‐partum. At 18 months post‐partum, the median UIC was similar to the level measured during pregnancy. The stability coefficient in tracking analyses of UIC was at its lowermost between 6–12 and 12–18 months post‐partum. Given the large interindividual and intraindividual variability of UIC, a high stability coefficient was not expected. The very low stability from 6 to 18 months post‐partum may be partially explained by a change in breastfeeding status for many at various time points during this period. Women who exclude milk and dairy products from their diets and/or do not use iodine‐containing supplements may be at risk of iodine deficiency and may possibly have an increased risk of thyroid dysfunction and conceiving children with nonoptimal developmental status. The current study results underline the importance of implementing actions to improve iodine nutrition among pregnant and lactating women in Norway.

## CONFLICTS OF INTEREST

The authors declare that they have no conflicts of interest.

## CONTRIBUTIONS

LD, MK, VM, LS and MWM planned and conducted the study. IA, MSM and SH planned the paper draft. IA and MSM did the statistical analyses and wrote the first draft of the manuscript. All authors edited the manuscript.

## Supporting information

Appendix S1: Number of participants and median (min‐max) gestational week/weeks post‐partum in the different data collection waves in the LiN study.

Appendix S2: Urinary iodine concentrations in 18 months old children and their mothers 18 months post‐partum (*n* = 386), with fitted line. Participants with values above 500 μg/L are not shown (children *n* = 4, mothers *n* = 2) due to visuality.
