# Supplementary material for: Iodine status during pregnancy and at 6 weeks, 6, 12 and 18 months post‐partum
Source: Matern Child Nutr. 2020 Jun 29;17(1):e13050. doi: 10.1111/mcn.13050 (PMC7729798; doi:10.1111/mcn.13050)
Supplement: Supplementary file 1 — Appendix S1: Number of participants and median (min‐max) gestational week/weeks post‐partum in the different data collection waves in the LiN study. [file MCN-17-e13050-s001.docx]

All participants in the Little in Norway (LiN) cohort study (n=1036) pregnant women

**Wave 1: Pregnancy**

Provided urine sample: n=1004, median (range) gestational week: 24 (9-34)

**Wave 2: 6 weeks post-partum**

Provided urine sample n=915, median (range) postnatal week: 7 (2-21)

**Wave 3: 6 months post-partum**

Provided urine sample n=849, median (range) postnatal week: 26 (19-36)

**Wave 4: 12 months post-partum**

Provided urine sample n=733, median (range) postnatal week: 52 (44-67)

**Wave 5: 18 months post-partum**

Provided urine sample n=714, median (range) postnatal week: 79 (72-96)

**Supplementary appendix 1**: Number of participants and median (min-max) gestational week/ weeks post-partum in the different data collection waves in the LiN study.
