# Supplementary material for: Iodine status during pregnancy and at 6 weeks, 6, 12 and 18 months post‐partum
Source: Matern Child Nutr. 2020 Jun 29;17(1):e13050. doi: 10.1111/mcn.13050 (PMC7729798; doi:10.1111/mcn.13050)
Supplement: Supplementary file 2 — Appendix S2: Urinary iodine concentrations in 18 months old children and their mothers 18 months post‐partum (n = 386), with fitted line. Participants with values above 500 μg/L are not shown (children n = 4, mothers n = 2) due to visuality. [file MCN-17-e13050-s002.docx]

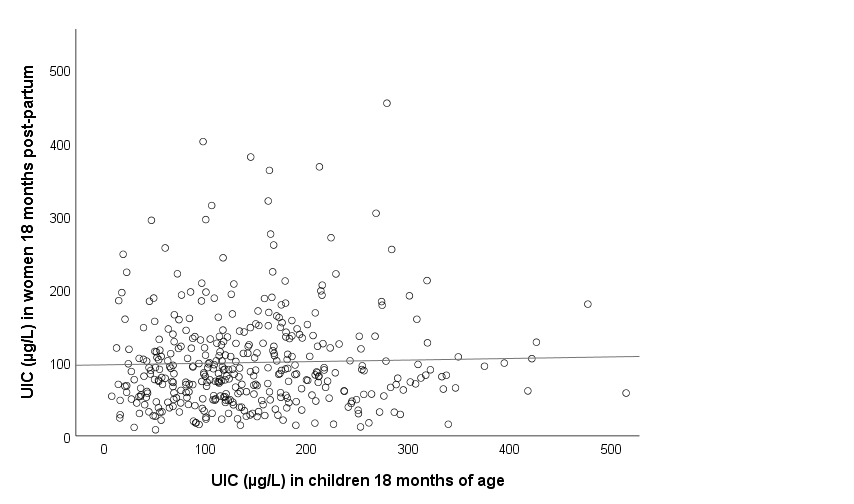
**Supplementary Appendix 2**: Urinary iodine concentrations in 18 months old children and their mothers 18 months post-partum (n=386), with fitted line. Participants with values above 500 µg/L are not shown (children *n*=4, mothers *n*=2) due to visuality.

.
